# What Are the Barriers Impeding Female Graduates in Higher Education from Pursuing Entrepreneurship in China? An Investigation from a Theory of Planned Behaviour Perspective

**DOI:** 10.3390/bs14080651

**Published:** 2024-07-27

**Authors:** Zhixiu Chen, Wuyuan Guo

**Affiliations:** 1 Faculty of Education and Society, University College London, London WC1H 0AL, UK; zhixiu.chen.20@ucl.ac.uk; 2 Department of Seconday Education, The Education University of Hong Kong, 10 Lo Ping Road, Tai Po, New Territories, Hong Kong, China

**Keywords:** female entrepreneurship, higher education, theory of planned behaviour

## Abstract

This study investigates the obstacles encountered by female graduates in China’s higher education system when considering entrepreneurship by employing the Theory of Planned Behaviour as an analytical framework. Although entrepreneurship is widely acknowledged as crucial for economic and personal growth, gender inequalities remain, with women being severely underrepresented. The primary objective of this study is to gain insight into the underlying reasons behind the reluctance of female university students, specifically in China, to pursue entrepreneurial ventures. In this study, we conducted 30 semi-structured interviews with female university students from different majors to identify the key barriers that discourage them from starting their businesses. The factors discovered are instrumental and affective attitudes towards entrepreneurship, subjective norms (family, friends, teachers, and others), and perceived behavioural control (encompassing past experiences, second-hand information, and anticipated obstacles). The results showed that a conservative familial atmosphere, risk-averse cultural norms, and limited access to helpful resources and experiences in universities are major obstacles. This study enriches the present knowledge by providing a detailed explanation of the gender-specific obstacles in entrepreneurship in the Chinese context. It proposes that interventions at the educational and governmental levels are imperative to promote female entrepreneurship.

## 1. Introduction

Entrepreneurship is a multifaceted and dynamic concept that entails actively seeking opportunities, displaying innovation, and embracing risks in order to create value across various economic and social contexts [1]. While entrepreneurship significantly enhances economic growth and promotes personal and societal development [2,3], it is also subject to persistent gender inequities. Women are notably underrepresented in entrepreneurial activities and face unique challenges that are often overlooked in the entrepreneurship literature [4,5]. Many studies have evidenced that higher education plays a key role in fostering entrepreneurial intentions and actions [6,7,8]. Therefore, it is worthwhile to examine university students’ decisions and practices related to entrepreneurial careers. Multiple aspects such as economic policy, cultural background, family tradition, life plan, individual personality, etc., could facilitate or impede graduates to engage in entrepreneurship, which is worthy of further investigation where the collectivism value is salient like in China. Furthermore, previous research has predominantly emphasised the factors that motivate college students to initiate a business, while neglecting the factors that deter them from doing so, especially among female graduates [9]. To fill these gaps, this study aims to analyse the barriers impeding female graduates in higher education from pursuing entrepreneurship in China.

## 2. Literature Review

### 2.1. Entrepreneurship

Entrepreneurship is a complex and ever-changing notion that involves actively seeking opportunities, being innovative, and taking risks to generate value across different economic and social settings [1]. Entrepreneurship mostly entails recognising and capitalising on unmet needs in the market, typically motivated by an urge for independence and a determination for bringing changes [10]. 

Owing to its most salient impact on economic expansion, employment generation, and technical progress, entrepreneurship is widely recognised as a crucial factor for achieving success in contemporary society [2,11]. Indeed, with the escalating global unemployment rates, numerous governments are relying on entrepreneurial start-ups as a means of generating job opportunities [12,13]. Recent research also highlights the fact that entrepreneurship plays an important role in strengthening economic resilience [14]. It does so by fostering innovation and adaptability in the market, which are crucial during the recovery stages after global shocks like the COVID-19 pandemic [15].

On top of its economic implications, entrepreneurship holds significance for comprehensive personal development as entrepreneurship goes beyond simply starting new businesses by encompassing a mindset that flourishes with inventiveness, flexibility, and a readiness to navigate unpredictable situations [16]. Some scholars identify the wider socio-cultural effects of entrepreneurship given its power to promote diversity, foster a dynamic social climate, as well as contribute to sustainable development [3,17].

### 2.2. Entrepreneurship and Gender

Although the baseline requirements are equal for males and females to pursue entrepreneurship, the majority of investigations consistently affirm the existence of a disparity between men and women in terms of their engagement in entrepreneurial activities, entrepreneurial orientation, and the inclination to pursue entrepreneurship [18,19]. The 2023 Women’s Entrepreneurship Report [20] indicates that globally, approximately one in six women express an intention to start a business, a rate that is lower than the one in five men who report similar intentions. For actual start-up activities, there are 0.80 women entrepreneurs for every man engaged in such ventures. Even for those women who become entrepreneurs, research conducted across different countries reveals that they experience a significantly lower rate of objective success than male counterparts, characterised by slower business growth rates, less sales, and lower profitability [21,22]. The low willingness to engage in business and the greater difficulties experienced by women entrepreneurs are due to prevalent gender stereotypes [23,24]. Research further confirms the widely held belief that becoming an entrepreneur is predominantly associated with masculinity in society [25,26]. Although the proportion of female entrepreneurs is relatively small compared to males, research demonstrates that business females make substantial contributions to the entrepreneurship field [27] but their economic, socio-cultural, and other dynamic values are easily diminished [28]. Hence, the gender imbalance within entrepreneurship deserves greater academic investigation.

### 2.3. Entrepreneurship and Higher Education

Education, especially higher education, is crucial for cultivating entrepreneurship [6]. A UK-based longitudinal study discovered that universities have a vital role in offering entrepreneurial knowledge, instilling intentions, encouraging innovations, and nurturing entrepreneurship [7]. Over the past decades, universities have significantly expanded their role in promoting entrepreneurship, resulting in the emergence of new teaching methods, business cultures, and departments and programmes [29,30]. The emergence of the entrepreneurial university has superseded the traditional university model [31]. Universities have been embracing innovative educational strategies in entrepreneurship, such as the integration of business simulations, development of entrepreneurship curriculum, provision of extracurricular support activities, hosting seminars [32], and offering training programs [32,33,34,35,36]. Additionally, universities are adopting business plan competitions and grants as part of their entrepreneurial support framework for students [37]. Under such education, college students can acquire the entrepreneurship ability to recognise and resolve issues, collaborate in groups, assess risks, and skilfully communicate with people from various fields to be better equipped to become successful entrepreneurs [38].

Despite the extensive focus on fostering entrepreneurship by universities and the government, studies have shown that only a small proportion of graduates opt to initiate entrepreneurial ventures either during or shortly after graduation [39,40]. Considering the substantial investments made by universities and the consistently low proportion of students who become entrepreneurs so they would be limited in making contributions to society, it is equally worthwhile to explore the factors that drive graduates to pursue their businesses and the factors that deter them from doing so.

### 2.4. Entrepreneurship in China

Chinese higher education has made remarkable progress in increasing access to a large portion of the population since 1999 [41]. Nevertheless, the annual influx of millions of graduates, combined with the disparity between university curriculum and the demands of the labour market, results in a growing unemployment rate [42]. Over the past decades, the government has regarded entrepreneurship as an effective solution to addressing unemployment issues among university graduates [9] by issuing multiple policies to encourage innovation and entrepreneurship among young people and urges universities to offer essential assistance for students engaging in entrepreneurship [43].

With government promotion, Chinese universities have actively pursued innovation and entrepreneurship education through various means such as establishing institutions, organising lectures, recruiting professionals, hosting innovation and entrepreneurship competitions, offering training programmes, providing practical resources, and offering financial support [44]. After years of development, comprehensive educational resources and various instructional methods were employed to facilitate students in gaining a greater understanding of entrepreneurship [45].

Consequently, an increasing number of Chinese university graduates are considering entrepreneurship as a viable career choice [46]. The 2021 China College Students Entrepreneurship Report reveals a consistent increase in students’ intention towards entrepreneurship over the past five years. Approximately 96.1% of students have thought about starting a business, while 14% have already initiated or are in the process of planning a business venture [40].

Nevertheless, university graduates continue to encounter obstacles in the process of transitioning from their intention to actually starting their businesses [9]. Research indicates that Chinese university graduates do not consider self-employment and entrepreneurship as desirable occupations, despite the legislative incentives provided by the government and the intense competition in the job market [42]. The Report on Employment of Chinese College Students published by the Mycos Research Institute [47] shows that the self-employment rate among Chinese university students is not promising. The percentage of students engaging in independent business immediately after graduation has declined from 1.8% in 2018 to 1.2% in 2022, which remains a substantial disparity with graduates in Europe and America [43]. In the context of China, cultural influences are one of the underlying factors that exert substantial impact on students’ entrepreneurial intentions. According to Yang et al. [48], the prominent focus on collectivism in Chinese culture cultivates a preference for stable and safe career trajectories rather than the unpredictable nature of entrepreneurial endeavours. The presence of traditional gender roles further complicates this situation, as women are frequently anticipated to prioritise family obligations, which hinders their involvement in business [49]. Moreover, the influence of family, friends, and cultural norms on career choices is more significant in China when compared to cultures that value individualism, adding another layer of complexity to entrepreneurial intentions [50,51].

In light of these cultural influences as well as other factors, China has a significant population of university graduates who do not engage in entrepreneurship, with a notable proportion of females. Hence, it is vital to explore the factors that lead to the unwillingness of female graduates to enter the entrepreneurial world. Nevertheless, empirical research has predominantly examined the factors contributing to university graduates’ engagement in entrepreneurial activities, so there is an absence of literature that specifically investigates the factors hindering educated female youth from participating in entrepreneurship [9]. In addition, even though women constitute a significant ratio of potential entrepreneurs, there has been insufficient attention given to gender-specific perspectives on entrepreneurship [52]. Furthermore, although the topic of females’ entrepreneurship has been examined in Western literature, it has received comparatively less attention in China. Chinese female university students possess distinct cultural and societal backgrounds compared to those in the West, necessitating an in-depth and comprehensive exploration. To fill these research gaps, this study targeted investigating barriers hindering the entrepreneurial behaviours of female university graduates in the Chinese context.

### 2.5. Theoretical Framework

The Theory of Planned Behaviours (TPB) is a robust model for forecasting human behaviours [53], particularly when it comes to explaining planned actions such as the decision not to engage in entrepreneurship [54,55]. Therefore, the current study selected the TPB as the theoretical framework.

The TPB, developed by Ajzen [53,56,57], posits that a significant portion of human activity is influenced by conscious decision making and planning. According to this theory, behaviour is shaped by salient information or beliefs relating to actions. Behavioural intentions anticipate the actual actions taken. Simultaneously, intention is influenced by three primary antecedents: attitude towards behaviour, subjective norm, and perceived behaviour control. Attitude towards behaviour means an individual’s positive or negative assessment of the behaviour, showing the degree to which they hold a favourable or unfavourable position towards it. Attitude can be classified into two types: instrumental attitude, which refers to the inclination to engage in a specific activity, and affective attitude, which relates to the emotional inclination to refrain from performing the behaviour [56]. A subjective norm refers to the social pressures from important others received by a person to either engage in or avoid a specific behaviour. It can be separated into two types: injunctive and descriptive [58]. Injunctive normative beliefs relate to the expectation or subjective likelihood that a particular individual or reference group approves or disapproves of engaging in a specific behaviour. Descriptive normative beliefs involve individuals’ perceptions of the behaviour displayed by influential others. Perceived behaviour control is an individual’s perception of the level of difficulty associated with performing the desired behaviour [53]. Control beliefs can be formed by past experiences, indirect information, and contextual circumstances that can either increase or decrease the perceived degree of difficulty involved with the action. Generally, an individual’s intention to perform a behaviour is influenced by their attitude, subjective norm, and perceived behavioural control. The more positive the attitude and subjective norm and the higher the perceived behavioural control, the stronger the intention to perform a behaviour.

**Figure 1:**
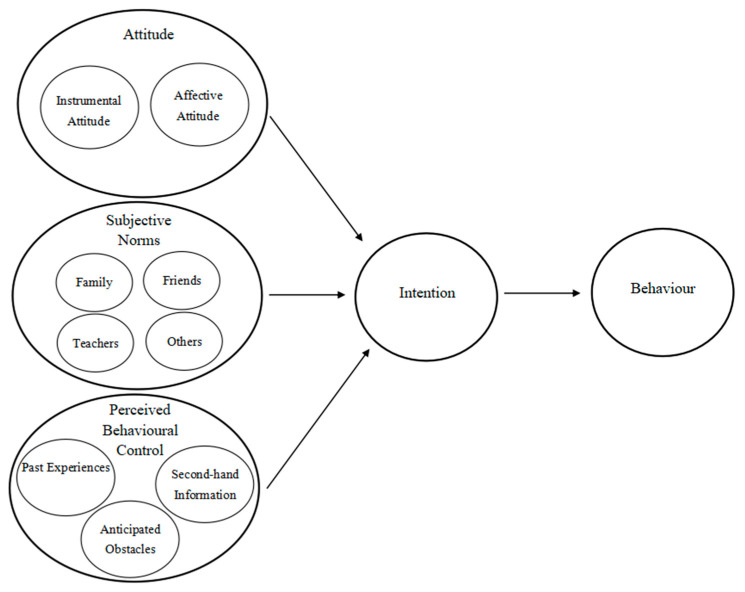
The theoretical framework of this study.

This study investigates the factors leading to the reluctance of female university students to initiate their own enterprises. As Figure 1 shows, the behaviour under examination is the decision to refrain from starting a business, while the intention refers to their unwillingness to engage in entrepreneurial activities. Based on the TPB, attitude towards behaviour is divided into instrumental attitude, which refers to the instrumental advantages of not establishing a business, and affective attitude, which is the subjective preference for staying away from entrepreneurship. The subjective norm encompasses four groups of people, including family, friends, teachers, and others, who possess a strong connection with university students. Thus, their actions and perspectives have the potential to impact decisions made by the university students. Finally, the perceived behavioural control involves past experiences with entrepreneurship, second-hand information they acquired, and anticipated obstacles they envisage on the path to abstaining from entrepreneurship. Based on the TPB and background mentioned above, this study answers the following research question:

What are the factors impeding female graduates in higher education to start an entrepreneurship venture in China?

## 3. Methodology

### 3.1. Participants

Based on the research question, there were several criteria for samples: no entrepreneurial intention, female, university student, and located in China. Therefore, this study involved a sample of 30 female undergraduates from 25 different Chinese universities with age ranging from 21 to 23 and majoring in law, arts, engineering, finance, and information technology (see Table 1). This study employed a purposive sampling technique to specifically target a certain subset of the population, hence increasing the relevance and depth of the data collected for the research objectives. This approach facilitated a thorough investigation of the different views of female university students from various academic fields and geographical regions in China [59]. The selected age group is also relevant because it consists of students who are about to graduate and are currently making decisions about their future routes. This coincides with the study’s objective to investigate the intention towards entrepreneurship in terms of career orientation.

The rationale for adopting this sampling approach is based on the need to comprehend intricate social phenomena within a bounded system, as proposed by Flyvbjerg [60], who promotes case-based, contextual analysis in social science research. The study achieves theoretical saturation by concentrating on a well-defined and informed sample. This ensures that the acquired data are representative and can potentially be generalised within the defined scope of the study [61].

### 3.2. Data Collection

Under each participant’s consent, 30 sets of 45 min individual semi-structured interviews were conducted via Zoom. The interview protocol comprised 25 interview questions according to the TPB, which can be divided into five parts: abstaining from entrepreneurship behaviours, attitude, subjective norms, perceived behavioural control, and intentions (see Appendix A). The interview data were audio-recorded and then transcribed into Chinese. Semi-structured interviews are crucial for examining the entrepreneurial intentions of university students because they enable a thorough investigation of personal views while maintaining the adaptability to accommodate emerging insights [62,63]. This approach supports the collection of comprehensive, detailed data essential for discerning the subtle motivational elements that affect students [64].

### 3.3. Data Analysis

Before analysing the data from the transcriptions, the accuracy of the transcriptions was verified by a comparison of the recordings with the transcribed texts. Only the cited answers from participants were translated into English for this manuscript.

Thematic analysis is a qualitative research technique employed to uncover, assess, and report on themes within collected data. It is highly efficient at organising intricate information into clear and separate topics [65]. Given the well-designed theoretical framework, the 30 collected sets of interview data were subjected to deductive thematic analysis in order to identify and present the underlying themes [66]. As delineated in Figure 1, the thematic codes were primarily classified into 5 themes with 10 sub-themes based on the TPB. By employing deductive thematic analysis via the lens of the TPB, this approach classifies data into predetermined themes systematically, hence improving the precision and depth of the study. This approach ensures that the results are well correlated with theoretical concepts, offering perceptive explanations of the respondents’ intentions [67].

## 4. Findings

### 4.1. Behaviours

Regarding the present study, the specific behaviours interviewees exhibited are the choices they made on whether to start up their own business after graduation. All interviewees explicitly expressed that they were not going to initiate an enterprise after completing their undergraduate degree. The majority of interviewees prepared to pursue postgraduate study, while some were engaged in job applications.

### 4.2. Intentions

The participants were requested to provide a rating about their intention of avoiding initiating a business. The results indicate that the vast majority of participants, with the exception of two who evaluated their intention as 3, provided scores of 5 or above. Specifically, eight participants scored 10, eight scored 9, five scored 8, five scored 7, and two scored 5. These scores show the obvious reluctance of participants in engaging in entrepreneurship. As Ding said,

“I have no plans to start a business at all. I am actively seeking an internship, and I am preparing for grad school at the same time.”

—Ding

Regarding her post-graduation plans, Ding noted that she is simultaneously preparing two different paths, but she has no intention of initiating an enterprise.

### 4.3. Attitudes

According to Ajzen [53], one of the determinants of intention in the TPB is the attitude toward the behaviour. It describes an individual’s evaluation or appraisal of the behaviour in question, indicating the extent to which they have a favourable or unfavourable stance towards it. Attitude can be categorised into two types: instrumental attitude, referring to the inclination to enacting a certain behaviour, and affective attitude, referring to the emotional disposition towards performing the behaviour [56].

#### 4.3.1. Instrumental Attitude

More than half of the interviewees (n = 16) noted that refraining from entrepreneurship could result in greater concentration on passing their postgraduate examinations or seeking jobs. Taking Han as an example,

“Because my goal is clear, I intend to become a teacher after passing teaching test. If I don’t start a business, I can concentrate on studying for the exam without having to spend a lot of time and energy on my business.”

—Han

In Han’s response, entrepreneurship is perceived as a divergence from her life goals, thereby being regarded as an ineffective use of time and effort.

In addition, 11 participants indicated that not embarking on entrepreneurial endeavours is a means to mitigate the potential risks of business failure. Also, ten participants agreed that choosing not to start a business at present will alleviate financial burdens and competitive pressures. As Yu mentioned,

“As a design major, the costs for materials and other essentials are quite high. Consequently, my parents provided me with a substantial allowance. Continuing my education at the postgraduate level will allow me to maintain the financial support I receive from my parents without worries.”

—Yu

In Yu’s view, opting for postgraduate studies instead of pursuing entrepreneurship provides her with a sense of comfort as she can rely on monetary support from parents, thereby mitigating potential financial stress.

Moreover, nine participants displayed the view that acquiring experience and knowledge at the present stage is essential prior to considering entrepreneurial pursuits. Furthermore, seven interviewees suggested that not starting a business serves as a way of maintaining their desired level of stability in life. They could have more leisure time and freedom compared to entrepreneurs. Other instrumental elements including the avoidance of parental nagging and a broadening of life’s possibilities through interests or career exploration were also mentioned by participants.

#### 4.3.2. Affective Attitude

By rating emotional preference towards not conducting entrepreneurship, all interviewees indicated that their preference for not starting a business was at a minimum of 5 on a scale of 1 to 10. Notably, 15 out of 30 interviewees gave a rating of 8. The data show that the behaviour of not starting a business is likely to be linked to emotional dispositions, and there is no case in which individuals possess a genuine desire to start a business but opt not to pursue it.

Regarding the reasons why they do not like entrepreneurship (Table 2), more than half of the interviewees (n = 17) emphasised that psychological distress is one of the most important factors they consider. They define the state of not starting a business as their comfort zone, feeling more relaxed and less anxious compared to starting a business. Take Ling as an example:

“I find studying in school to be my comfort zone. The psychological pressure is not overwhelming, and it helps me avoid internal conflicts since I do not constantly worry about how my business are going to develop every day.”

—Ling

According to Ling, the current state of studying is a familiar and manageable pattern of behaviour. However, engaging in new behaviour, initiating a business venture, might induce significant psychological strain due to apprehension regarding the unpredictable trajectory of the enterprise.

In relation to the shift in attitudes towards not participating in entrepreneurial pursuits, it was found that 12 interviewees initially held negative views towards not starting a business in their childhood but currently perceive it as favourable. Conversely, eight interviewees expressed positive sentiments towards avoiding starting a business before they entered higher education but now exhibit diminished agreement. Additionally, ten interviewees reported that their perspective on it has remained unchanged since their early years.

### 4.4. Subjective Norms

The second predictor of intention in the TPB, as proposed by [53], is a social factor known as subjective norms. It refers to the perceived social pressure exerted on an individual to engage in a particular behaviour. There are two types of normative belief: injunctive and descriptive [58]. According to Ajzen [57], injunctive normative beliefs refer to the anticipation or subjective possibility that a certain individual or group of reference supports or opposes the performance of the specific behaviour under consideration. Descriptive normative beliefs relate to individuals’ beliefs regarding the behaviour exhibited by significant others. Within the scope of this study, the referent people of interviewees include family, friends, teachers, and other relevant parties, as summarised in Table 3.

#### 4.4.1. Family

The majority of participants (n = 26) indicated that their family atmosphere impacted their intention of not choosing to start a business. Their family atmosphere generally showed a conservative tendency, characterised by a reluctance to engage in risk taking behaviours. A significant number of participants (n = 20) reported that their parents displayed a preference for them to be permanently employed by the government. Take Zhang and Huang as examples:

“My parents believe that starting a business can be quite challenging, especially for a girl. So they think it may be more suitable for me to get a job within government departments, as it would provide a more stable ways of life.”

—Zhang

“As an only child and daughter, my parents have always wanted me to have a stable life and avoid significant risks. When I was young, they’ve already planned for me to pursue a degree in transportation and eventually work for the national transportation bureau.”

—Huang

As Zhang and Huang expressed, their parents revealed a tendency to encourage the pursuit of low-risk occupations, with gender being a contributing factor in their decision making process. This was very common among the parents of the interviewees, with 20 interviewees’ parents expressing that due to the interviewees’ gender, “settling down for life” was the most desirable future they had planned for their daughters. It is clear that starting up a business is rarely seen as an avenue of achieving long-term stability and security.

In addition, the majority of interviewees (n = 23) noted that their parents’ occupation was also likely to influence their intention. Interviewees whose parents were employed by the government showed an affinity for pursuing secure jobs akin to their parents. Ying provided an explanation for the underlying cause of this phenomena.

“Both of my parents are government employees. Observing their lifestyle, it seems that they have achieved a comfortable life without excessive effort. I think I can be happy by pursuing a similar career path, so I question the necessity of venturing into the unpredictable entrepreneurship and taking on the risks.”

—Ying

Ying’s response reveals her satisfaction with the current state of parents, therefore exhibiting a willingness to maintain a comfortable lifestyle by pursuing a career that aligns with that of her parents. In contrast, interviewees whose parents were entrepreneurs claimed that they had observed their parents’ arduous endeavours and thus desired to avoid similar hard work in their own careers. As Liang mentioned,

“My dad once started his own business, but it wasn’t very successful. He established an environmental company, which required him to travel all over the country, often to quite remote areas. It seemed like a tough and exhausting experience. So, I don’t want to go through the same hardships as he did.”

As Liang expressed, witnessing her father’s arduous yet ultimately fruitless entrepreneurial attempt instilled in her a sense of fear towards the difficult process of entrepreneurship, causing her to be hesitant to pursue entrepreneurship like her father.

Furthermore, the interviewees identified an authoritarian parenting style (n = 12) and insufficient family resources (n = 6) as factors that influence their decision not to pursue entrepreneurship.

#### 4.4.2. Friends

All participants showed that they were subject to varied degrees of influence from their friends. This impact can be categorised into three types: surrounding atmosphere, friends’ experiences, and friends’ judgement. Of these, surrounding atmosphere was the most frequently cited factor (n = 24), followed closely by friends’ experiences (n = 15). A few interviewees also mentioned friends’ judgement (n = 3).

Primarily, surrounding atmosphere influences the intention of interviewees imperceptibly. Take Zhou as an example:

“Like me, my friends majored in clinical medicine and had defined career trajectories. They all intended to become doctors in large hospitals and had not thought to establishing their own businesses.”

—Zhou

Like Zhou, university students often tend to form social networks mostly composed of peers within the same majors. Consequently, it may lead to a greater homogeneity in future career trajectories, and these networks may influence one’s entrepreneurial desires.

Additionally, friends’ experiences also play an important role in influencing their intentions. Some interviewees referenced the entrepreneurial attempts of their friends. It is noteworthy that both successful and unsuccessful entrepreneurial experiences contributed to the respondents’ low willingness to start their own business. As Ye and Meng mentioned,

“I find myself greatly being influenced by the experiences of my friends who have faced setbacks in their entrepreneurial pursuits. Observing their challenges and shortcomings, I draw parallels to my capabilities and identify similar issues within myself, such as lacking entrepreneurial skills, not being ambitious enough, and being disconnected from business facets of society.”

—Ye

“My roommate started an online clothing store. She sources cheap clothes from wholesale markets, models them herself in attractive photos, and then sells them at a higher price. I think she has an incredible talent for entrepreneurship. I would never have thought of doing something like that.”

—Meng

Based on Ye’s answer, the unsuccessful entrepreneurial endeavours of her friends prompted her to assess her own competencies, leading her to conclude that she lacked the requisite aptitude to initiate a business and thus give up. On the other hand, as Meng responded, the presence of successful entrepreneurial experiences among friends also appears to prompt interviewees to engage in self-reflection, leading them to perceive themselves as less talented or capable than those friends.

#### 4.4.3. Teachers

In the college lives of students, teachers often act as frequent points of contact. With the exclusion of two, the majority of participants (n = 28) indicated that teachers impacted their intention to not choose to be entrepreneurs. The surrounding atmosphere (n = 14) shaped by teachers was considered as the most contributing factor according to interviewees’ responses. As Pan said,

“I feel that teachers in China tend to tell students to avoid risks from a young age. Additionally, the whole education system lacks a sufficient emphasis on career-oriented education, resulting in a limited understanding of entrepreneurship among students. Moreover, there is a prevailing belief that only those with poor academic credentials would choose to launch own business to earn a living.”

—Pan

Pan highlighted two key perspectives regarding the teaching philosophy of teachers and the overall Chinese education system. Firstly, she observed that teachers often prioritise risk avoidance when instructing students. Secondly, she noted that the macro-level deficiency in career-related education within the Chinese education system has resulted in a limited understanding of entrepreneurship among students, consequently leading to a scarcity of students opting for entrepreneurship as a viable career path. She also highlighted the presence of a stereotyped bias against entrepreneurs throughout society, which may partly contribute to the inclination of contemporary students to not pursue entrepreneurship.

Furthermore, the interviewees (n = 13) frequently highlighted the limited availability of supporting resources offered by teachers and schools. Interviewees stated that they do not have many resources available that deal with entrepreneurship; instead, their world is full of postgraduate opportunities and job postings. As Qian responded,

“The university claims it offers subsidies to students who start their own businesses, but no one has explained how to actually apply for these subsidies. Additionally, university does offer entrepreneurship courses, but teachers only teach some theoretical content. There aren’t any practical lessons that teach starting a business step by step. However, when it comes to job-searching courses, teachers often provide a lot of support and information, such as how to revise your CV and how to perform in interviews.”

In addition, direct suggestions provided by teachers have an impact on intention. Several interviewees (n = 10) indicated that teachers provided them with explicit recommendations regarding their career paths, which consistently discouraged them from pursuing entrepreneurship. Take Wang’s sharing, for instance:

“My university counsellor advised girls with pursuing graduate school, finding a good husband, and subsequently working as school counsellors was deemed as the best life plan. Just like her.”

—Wang

Wang’s response reflects that university counsellors provide students with direct guidance on career choices and life planning, drawing from their personal experiences. It is worth noting that the advice provided has an obvious presence of gender stereotypes.

Furthermore, some participants (n = 5) perceived the experiences of teachers, especially unsuccessful entrepreneurial attempts, as an important aspect influencing their intention.

#### 4.4.4. Others

In addition to the three aforementioned groups as subjective norms, participants also identified other people who had influenced their intention. These people can be broadly classified into three types: non-entrepreneurs, successful entrepreneurs, and unsuccessful entrepreneurs. Among them, those whom participants talked about the most were the successful entrepreneurs they encountered. By comparison, eight participants found a huge disparity between their own abilities and those possessed by accomplished entrepreneurs. Consequently, participants realised that they lacked the necessary attributes to achieve success in the field of business, leading them to dismiss the notion of beginning on their own businesses. Take Xie as an example:

“I met several alumni who are founders of an AI-generated image company which has gained significant attention this year. I was surprised that the company was established by only three people. I began to doubt my capabilities. I questioned whether I am truly qualified to start a business, particularly within a small company where everyone carries significant responsibilities. It is so stressful.”

—Xie

From Xie’s perspective, upon gaining insights into the experiences of successful entrepreneurs, she became aware of the substantial disparity between her own conditions and those of the entrepreneurs. The resultant sense of pressure stemming from this comparison ultimately discouraged her from pursuing business. In addition, six interviewees acknowledged that the experiences of successful entrepreneurs, such as the trials and tribulations at the early stage, prevented them from pursuing a similar trajectory.

Interviewees became less likely to launch a firm after learning about the failures of other entrepreneurs, in addition to successful ones. Take Lu as an example:

“I know a senior who used to start his own business in after-school tutoring. Unfortunately, his venture was cut short due to the implementation of national policies about banning the entire tutoring industry. His business took a direct hit. Policies are always unpredictable. He is a very hardworking person, but his business still failed completely, making me feel that it is so risky.”

—Lu

According to Lu, despite exerting significant effort, entrepreneurs are nevertheless susceptible to the danger of business failure when confronted with unfamiliar variables. As a result, the interviewees had a heightened perspective of the risks associated with entrepreneurship.

### 4.5. Perceived Behaviour Control

Ajzen [53] posits that the achievement of desired behavioural outcomes is influenced by the availability of resources and opportunities. The investigation of the perceived behaviour control and its subsequent impact on intentions and behaviours is of more psychological interest than that of actual control. The perceived behaviour control refers to an individual’s subjective evaluation of the level of ease or difficulty associated with engaging in a particular behaviour. Control beliefs can be shaped by previous experiences, second-hand information, and contextual factors that can shape the perceived level of difficulty associated with the activity.

#### 4.5.1. Past Experiences

With the exclusion of seven, the majority of the participants (n = 23) lacked previous experience in initiating an enterprise. The seven interviewees provided accounts of instances in which they experienced failure in their attempts to start their business or discontinued their efforts prematurely. In Zhuo’s words,

“I co-owned a milk tea shop with a friend in the past. It is really hard so we closed down that shop after six months. The primary reason was the significant pressure and persistent frustration stemming from my excessively high expectations. I had anticipated substantial sales, but the actual outcome was disappointing. I felt that I was getting farther away from my ideal, and I could not stand the feeling of falling short.”

—Zhuo

Zhuo recalled her unsuccessful attempt to establish her business and concluded that the discrepancy between her ideal and actual circumstances caused her to perceive a loss of control regarding the business, ultimately leading to its termination.

In contrast to Zhuo, the majority of participants had never established their own enterprise. However, a significant number of them possessed an idea for business plan, albeit unimplemented, due to limited resources and opportunity. Take Zheng as an example:

“I have considered setting up a stall in the school. My roommate suggested that it could be a profitable idea. I didn’t implement it because I was unsure about the legality of setting up a stall and concerned about potential gossip from other students. Besides, my roommate went home for every weekend, so I didn’t have partner to go with.”

—Zheng

Despite being encouraged by friends and harbouring a positive outlook on the potential advantages of entrepreneurship, Zheng ultimately chose not to pursue her own business. This decision was influenced by a limited comprehension of pertinent legal regulations, apprehension regarding societal perceptions, and the absence of a suitable collaborative partner. In this case, maintaining her previous lifestyle was much safer than engaging in a new business.

#### 4.5.2. Second-Hand Information

The increasing prevalence of contemporary technology has led to a growing dependence on the internet as the main source of second-hand information. This part explores second-hand information obtained from TV and the internet which contributed to the respondents’ intentions not to start their own businesses. More than half of the participants (n = 18) believed that news they saw online made a significant contribution. As Shao said,

“According to the news I’ve been following online, a significant number of people experienced bankruptcy due to the three-year COVID-19 pandemic. Based on my reading of these news, I think starting a business is so risky.”

—Shao

After reading abundant news, Shao developed a heightened awareness of the potential risks faced by firms and therefore developed apprehension against engaging in entrepreneurial activities. Likewise, many interviewers concurred that news articles, particularly those related to bankruptcy during the COVID-19 pandemic, left a profound impression on them.

In addition, nine interviewees mentioned that their willingness to not start a business became stronger after being exposed to the arduous entrepreneurial journeys shared by internet influencers or the sudden decline in the popularity of certain internet influencers. According to Wei,

“I used to enjoy watching Li Ziqi’s videos. She often shared her idyllic rural life and accumulated billions of followers. However, one day she suddenly stopped updating, and her popularity plummeted. I feel that starting a business in social media is too exhausting. You have to continuously produce new content every day; otherwise, you risk losing your audience quickly.”

This case gave Wei an in-depth awareness that entrepreneurship is challenging and carries the inherent risk of failure at any time. Moreover, five interviewees said that TV series or documentaries featuring other entrepreneurs’ experiences had instilled in them a sense of admiration but fear of entrepreneurship.

To sum up, by observing various second-hand information or stories regarding entrepreneurship, respondents realised their preference for a life characterised by stability and tranquillity, since such a life is more within their control. They were reluctant to confront the adverse consequences of initiating a business that were entirely beyond their control.

#### 4.5.3. Anticipated Obstacles

Referring to the anticipated obstacles of not starting a business, interviewees’ answers concentrated on resulting in a monotonous future (n = 18). As Lin mentioned,

“I feel like the days stretch out endlessly before me, monotonous and lacking any sense of excitement or novelty.”

—Lin

From Lin’s viewpoint, entrepreneurship encompasses elements of novelty, enjoyment, and creativity, whereas opting not to start a business implies a rather boring life. In addition, ten participants stated that not starting their own business prevented them from challenging themselves and realizing their life values. As Ren mentioned,

“Starting a business might keep me energetic and enthusiastic because I need to concentrate fully on facing every challenge. If I choose not to start up a business, I may lose such youthful courage to challenge myself and getting self-actualisation.”

Also, seven participants highlighted that their decision could potentially result in the loss of lucrative prospects. Moreover, four participants thought that their decision might lead to a missed chance to engage in networking with accomplished people. However, four interviewees declared that they had no regrets or obstacles regarding this decision.

This part explains why all participants did not act to initiate a business but most of them did not assign a full score to their level of intention. This is due to their desire for the potential advantages of entrepreneurship, but ultimately they chose not to initiate a business in light of the greater drawbacks associated with entrepreneurship.

To sum up, the female college students’ reasons for not pursuing entrepreneurship in terms of the perceived behaviour control factor are organised in Table 4.

## 5. Discussion

This study interviewed 30 Chinese female undergraduates to explore the reasons behind non-entrepreneurial behaviours from the perspective of the TPB, upon which the following discussion will be unfolded.

The first influential factor in the TPB is attitude. which consists of instrumental and affective attitude. Regarding instrumental attitude, the research reveals that participants considered not pursuing entrepreneurship as a strategic choice that aligned with their priorities, such as focusing on postgraduate exams or job hunting. Similarly, Hjeltnes et al. [68] discovered that maintaining concentration is crucial for university students, particularly during periods of pressure. Hence, final-year students experiencing transition stress are inclined to focus on a single goal, rather than contemplating entrepreneurship concurrently. Participants in this research also prioritise maintaining stability in finance and lifestyle, so entrepreneurship is regarded as an uncertain factor they try to avoid. However, some scholars believe that university students are more prone to undertaking risks and seeking new experiences compared to individuals at other stages of life [69,70]. This paradoxical phenomenon may be attributed to different contexts of countries. In the context of China, Li [71] highlights that the economic deceleration and heightened competitiveness in the labour market have instilled a profound perception of risk among undergraduates. Particularly in the aftermath of the societal instability caused by COVID-19, they favour stability and security. From a gender perspective, although the basic requirements for entrepreneurship are theoretically unbiased towards gender, there are still differences in the involvement, attitude, and inclination towards entrepreneurship between genders. This is because women are more likely to be strongly influenced by their perception of risk, which often deters them from pursuing entrepreneurial endeavours [19].

Referring to affective attitude, the findings indicate that participants uniformly displayed a minimum emotional preference score of 5 out of 10 against entrepreneurship as they linked their dislike to psychological distress, considering not starting a business as their comfort zone. The findings corroborate Duong et al.’s research [72], indicating that psychological stress has a detrimental impact on students’ attitudes towards entrepreneurship in higher education. Furthermore, Chadwick and Raver [73] emphasised the gender difference in psychological distress, suggesting that females may encounter greater levels of psychological distress in entrepreneurship.

According to the TPB, non-entrepreneurial actions are impacted by subjective norms encompassing family, friends, teachers, and others. The findings demonstrate that the family atmosphere has a substantial influence on participants’ intentions against entrepreneurship. Participants’ decisions were affected by a pervasive and conservative atmosphere characterised by risk aversion. Parents exhibited a desire for their daughters to be permanently employed by the government, the jobs of which are deemed stable and appropriate for girls. That implies a prevailing bias based on gender stereotypes. Many studies likewise show the importance of parents’ aspirations for children’s occupational paths in both Western and East Asian countries [74,75]. In China, scholars discovered that parents’ expectations have a greater impact on girls’ career choices than on boys’ [74]. Moreover, this research shows that participants’ career choices are significantly influenced by parental occupations. There is a tendency to follow in the steady footsteps of their parents and avoid pursuing unpredictable entrepreneurial endeavours. This behaviour is observed and elucidated by other research. Firstly, children are more familiar with their parents’ career compared with other fields of work [76]. Secondly, children may internalise occupationally related values from their parents [77]. A final reason is that children can acquire skills and knowledge by observing their parents’ work and listening to their parents’ experiences [78].

In addition, all participants unanimously recognised the impact of their friends. The surrounding atmosphere gradually influences the intents of individuals, especially among social networks that are centred around the same major in universities. Participants generally notice that the absence of an entrepreneur among their friends with the same major leads to a lack of enthusiasm for entrepreneurship. Indeed, other research findings have suggested a high correlation between entrepreneurial tendencies and friendships. According to Wongnaa and Seyram [79], the atmosphere shaped by friends can influence students’ decisions to graduate as entrepreneurs in higher education. Furthermore, current research presented that the entrepreneurial experiences of friends, regardless of their outcomes, also have a substantial influence on the participants’ intentions, leading to introspection regarding their own abilities and aspirations. Because peers act as influential role models of university students whom they spend the most time with, peers convey their own opinions and provide vital sources of information that students utilise to shape their perceptions of capabilities and attitudes [80]. In addition, Chi et al. [81] suggest that female millennials are easily impacted by their friends when it comes to decision making. Therefore, social networks may exert significant effects on young women’s choices about entrepreneurship.

Similar to friends, teachers are another group that routinely interacts with university students, so most students perceive teachers as significant influences on their career decisions, particularly discouraging entrepreneurship. Teachers commonly promote risk aversion and neglect career education, resulting in students having a poor comprehension of entrepreneurship. This is a prevalent concern in China. Chinese university teachers are reported to have passive attitudes and beliefs toward career education [82]. The current study discovered that teachers’ gender-biased advice and stereotypical views further deter students, which is echoed by Yang et al. [83], who demonstrated that gender stereotype has persisted as a problem among Chinese college students. The prevalence of gender-biased advice may have distinct effects on male and female students, dissuading females from pursuing entrepreneurship due to perceived risks and steering males towards subjects traditionally associated with masculinity. It is essential to confront these prejudices in career advising in order to promote gender equality and enhance the entrepreneurial ecosystem by embracing a wide range of talents and viewpoints.

In addition, participants’ entrepreneurial intentions are negatively influenced by observing other entrepreneurs. Successful entrepreneurs lead participants to realise qualities they lack and responsibilities they will bear, causing self-doubt. Unsuccessful entrepreneurs promote participants to recognise the unpredictability and risks in entrepreneurship. In contrast, Liu et al. [84] investigated 150 Chinese undergraduates and found that both successful stories and failure entrepreneurial stories positively affect participants’ perceived intentions. These two distinct findings may be caused by a shift in times. Before 2020, people had not seen the serious setback in business caused by COVID-19 and were therefore more likely to have a positive attitude towards the experience of entrepreneurs whether they succeeded or failed.

Furthermore, as stated in the TPB, students’ lack of intention towards entrepreneurship is determined by their perceived behaviour control, which includes their past experiences, second-hand information, and anticipated obstacles. When investigating past experiences, research showed that most participants lacked prior experience in business initiation. A few who attempted faced failures due to a loss of control over business. Others had business ideas but did not proceed, deterred by legal and social concerns and a lack of support. They believe that maintaining their current lifestyle is better under their control. Other studies reinforced that young people’s entrepreneurial intentions are impacted by their experiences attempting to start a business [85,86].

Participants’ perceptions of entrepreneurship have also been shaped by the accessed information. Most participants were discouraged from becoming entrepreneurs after receiving online news that emphasised the risks and failures of entrepreneurship, notably during the COVID-19 pandemic. Entrepreneurial stories on difficulty and unpredictability, depicted by social media, reinforced their apprehension. This led to a preference for a stable, controlled lifestyle over uncertain business ventures. Likewise, some scholars have noticed that mass media substantially influences university students’ career selections [87]. Social media usage heightens students’ anxiety regarding career prospects while simultaneously encouraging their career exploration [88]. Moreover, gender significantly influences how individuals perceive these controls, with female students being more inclined to be discouraged by the obstacles they perceive, as noted by Vamvaka et al. [89]. However, Lee et al. [90] argued that social media has less impact on students’ entrepreneurial intentions than traditional factors such as the influence of family and friends.

Despite not intending to start a business, participants expressed their anticipated regret and obstacles relating to this decision. They thought that forgoing entrepreneurship may lead to a lack of enthusiasm and novelty in life. Many participants associated entrepreneurship with personal fulfilment and potential financial gains. However, they ultimately decided against starting a business due to the significant disadvantages that come with entrepreneurship, outweighing these possible advantages. Other research explored young adults’ mechanism of career decision making, revealing that they exhibit higher levels of rationality and decisiveness than people assume [91,92]. It explains the phenomenon wherein participants acknowledge the beneficial effects of entrepreneurship yet deliberately opt to avoid engaging in it.

## 6. Limitations and Implications

There are some limitations in this study. The number of female undergraduates sampled is limited to thirty, so the generalizability of research findings to other counties should be made with caution. It might not fully capture the diversity of experiences and perspectives of female university students across the country. Moreover, given the cross-sectional nature of this study, the collected data only refer to the current situation of university undergraduates expected to graduate in 2024 who experienced three years of the unprecedented COVID-19 pandemic, which could not often happen to future college students. The influence of such historical and special experience may have a profound effect on the interviewed female undergraduates, which deserves extra background explanation. Future research should consider longitudinal studies to examine how entrepreneurial intentions evolve over time and in different post-pandemic contexts. Expanding the sample size and including participants from diverse regions within China can provide a more comprehensive understanding of the barriers and motivators for female entrepreneurship. Examining the influence of distinct cultural, economic, and social factors in different provinces may provide valuable insights into regional disparities and facilitate targeted policy interventions.

Theoretically, this research fills a research gap by providing a new theoretical framework and viewpoint on the factors contributing to the lack of entrepreneurship intention among female undergraduates in China. Practically, the findings highlight the need for educational policymakers to incorporate comprehensive entrepreneurship education into university curricula in order to provide students with essential skills and knowledge. It is important to have policies that support gender equality and tackle conventional gender norms that hinder women from engaging in entrepreneurship. Training programs that specifically target risk management, business planning, and leadership abilities have the potential to empower female students, enabling them to overcome obstacles and actively pursue entrepreneurial opportunities. In addition, promoting a cultural change that emphasises the importance of innovation and willingness to take risks, while also showcasing exemplary figures and mentors, can serve as a source of inspiration for female students and offer them tangible assistance. By addressing these implications, policymakers and educational institutions can promote gender equality in entrepreneurship, which will contribute to broader economic growth and innovation.

## 7. Conclusions

This study examined the various obstacles that prevent female undergraduates in China from pursuing entrepreneurship. Based upon the TPB as the framework and vivid semi-structured interview data with 30 female undergraduates, the research discovered that the entrepreneurial intentions of women receiving higher education were significantly influenced by instrumental and affective attitudes, subjective norms, and perceived behavioural control. The findings emphasised the influential role of surrounding people, experiences with entrepreneurship, insufficient resources, and outside information on changing intentions towards entrepreneurship. The presence of a conservative family atmosphere, along with the impact of peer and teacher norms, underscored a wider cultural and educational context that discourages female graduates from engaging in entrepreneurship.

This study enriches the TPB by situating it within the unique socio-cultural and educational environment of China, therefore adding to a deeper understanding of gender inequalities in entrepreneurship, and highlights the necessity for targeted interventions by educational institutions and policymakers to tackle the identified obstacles for female graduates’ difficulty in pursuing entrepreneurship. By building on this study, future researchers can conduct longitudinal studies that offer valuable insights into the progression of these attitudes and intentions over time, especially during the economic downturn following the end of COVID-19, and try to advance the removal of obstacles to female entrepreneurship, thus promoting a more inclusive entrepreneurial climate.

## Figures and Tables

**Table 1:** Demographic information of participants.

Participants	Gender	Age	Grade	Major	Place of University
Ding	Female	22	Fourth Year	Chemical Engineering and Process	Hangzhou
Li	Female	23	Fourth Year	English	Ganzhou
Lin	Female	21	Fourth Year	Economics	Shanghai
Han	Female	21	Fourth Year	Business English	Chongqing
Tang	Female	22	Fourth Year	Accounting	Nanjing
Wang	Female	22	Fourth Year	Law	Chongqing
Zhuang	Female	23	Fourth Year	Primary Education	Jinhua
Ge	Female	21	Fourth Year	Fashion and Costume Design	Hangzhou
Zhang	Female	22	Fourth Year	Light Source and Lighting	Dalian
Zhou	Female	22	Fourth Year	Clinical Medicine	Hangzhou
Ying	Female	21	Fourth Year	International Business Economics	Nningbo
Zheng	Female	22	Fourth Year	Software Engineering	Shanghai
Pan	Female	21	Fourth Year	Finance	Wenzhou
Zhao	Female	22	Fourth Year	Cyberspace Security	Beijing
Ye	Female	21	Fourth Year	Law	Qingdao
Lu	Female	22	Fourth Year	Civil Engineering, Water Resources, and Transport	Hangzhou
Zeng	Female	23	Fourth Year	English	Hangzhou
Ling	Female	22	Fourth Year	Chinese Language and Literature	Shanghai
Shao	Female	22	Fourth Year	Spanish	Shanghai
Wu	Female	22	Fourth Year	Optical and Electronic Information Engineering	Wuhan
Zhuo	Female	23	Fourth Year	Law	Jinzhong
Xie	Female	22	Fourth Year	Interactive Media Arts	Shanghai
Huang	Female	22	Fourth Year	Intelligent Transportation	Beijing
Liang	Female	21	Fourth Year	Business Management	Guangzhou
Chen	Female	22	Fourth Year	Economics	Kunming
Yu	Female	22	Fourth Year	Jewellery Design	Beijing
Ren	Female	23	Fourth Year	Accounting	Guangzhou
Meng	Female	22	Fourth Year	Architecture	Suzhou
Wei	Female	21	Fourth Year	Broadcasting	Chengdu
Qian	Female	22	Fourth Year	Software Engineering	Shenzhen

**Table 2:** Attitudes towards not starting a business.

Attitudes towards Not Starting a Business	
Instrumental attitude	Focus on academics/job seeking (n = 16) Risk avoidance (n = 11) Financial stability (n = 10) Experience and knowledge acquisition (n = 9) Life stability (n = 7) Avoidance of parental pressure (n = 3) Career exploration (n = 3)
Affective attitude	Emotional preference against entrepreneurship (n = 30) Psychological comfort zone (n = 17)

**Table 3:** Subjective norms towards not starting a business.

Subjective Norms towards Not Starting a Business	
Family	Conservative attitudes towards risk (n = 26) Parental occupation influence (n = 23) Preference for government jobs (n = 20) Gender-based career expectations (n = 20) Authoritarian parenting style (n = 12) Limited family resources for entrepreneurship (n = 6)
Friends	Surrounding atmosphere (n = 24) Friends’ entrepreneurial experiences (n = 15) Friends’ judgments (n = 3)
Teachers	Risk avoidance (n = 14) Limited support for entrepreneurship (n = 13) Lack of career-oriented education (n = 11) Direct career path recommendations (n = 10) Stereotyped biases against entrepreneurs (n = 8)
Others	Self-doubt and perceived inadequacy to be entrepreneurs (n = 8) Perceptions of business instability and risk (n = 6)

**Table 4:** Perceived behaviour control towards not pursuing entrepreneurship.

Perceived Behaviour Control towards Not Pursuing Entrepreneurship	
Past experiences	Lack of previous entrepreneurial experience (n = 23) Business failures leading to perceived loss of control (n = 7)
Second-hand information	Negative impact of news on business risks (n = 18) Stories from internet influencers and documentaries creating fear of failure (n = 9)
Anticipated obstacles	Monotony and lack of excitement (n = 18) Prevented realisation of life values (n = 10) Loss of lucrative opportunities (n = 7) Missed networking chances (n = 4)

## Data Availability

The data are available to share upon request from the first author by email.

## Appendix A. Interview Questions Outline

**Beginning**

- What do you consider to be entrepreneurship?
- At what point does a business operation qualify as entrepreneurship?

**Behaviours**

- Are you preparing to start your own business?
- What are you primarily doing at the moment? (e.g., Preparing for postgraduate exams, civil service exams, teacher qualification exams, etc.)

**Attitude towards the behaviour**

Instrumental attitude:

- What benefits or value do you see in not starting a business?
- What positive impacts has not choosing entrepreneurship had on you at present?

Affective attitude:

- Do you feel comfortable, at ease, or happy with your current state of not starting a business?
- Purely from a preference standpoint, on a scale of 0 to 10, how much do you like or aspire to your current state of not starting a business? (Follow-up: Is the remaining score due to a liking for entrepreneurship?)
- Has your preference or attitude towards not starting a business changed since you were young?

**Subjective norms**

Family:

- What is your parents’ attitude towards whether you start a business or not? (Follow-up: if they’ve mentioned it directly/your own feeling)
- What is your parents’ attitude towards others starting their own businesses?
- How do you think your parents have influenced your choice not to start a business?

Friends:

- What impact have your friends had on your choice not to start a business?
- Have you discussed not starting a business with your friends?

Teachers:

- How have your current university lecturers, tutors, or professors influenced your choice not to start a business?
- Apart from your current university teachers, have any other teachers (from past educational experiences or extracurricular education) directly or indirectly influenced your current decision not to start a business?

Others:

- Besides the aforementioned parents, friends, and teachers, who else has influenced your decision not to start a business?
- Has your current school organised any entrepreneurship-related activities (competitions, courses, workshops)?
- What kind of spirit do you think these activities convey? Have they influenced your views on entrepreneurship?
- Apart from your parents, have any other relatives directly or indirectly influenced your choice not to start a business?

**Perceived behavioural control**

Past experience:

- Have you ever tried starting a business before? (Running a small business/having business ideas)
- How has this experience affected your current decision not to start a business?

Second-hand information (Internet):

- Do you think the Internet has influenced your decision not to start a business to some extent?
- What information/news/events that you’ve observed indirectly have prompted you not to start a business?

Anticipated obstacles:

- If you never start a business in your lifetime, what regrets or losses do you think you might experience?

Other factors increasing or reducing perceived difficulty:

- Are there any other factors that have contributed to the losses associated with not starting a business?

**Intentions**

- So, you have no intention of starting a business?
- On a scale of 0 to 10, what is your intention not to start a business?
- Are these intentions solely based on the factors mentioned above, or are there other factors involved?
